# Impact of a pharmacy department–wide transitions-of-care program on inappropriate oral antibiotic prescribing at hospital discharge

**DOI:** 10.1017/ash.2022.327

**Published:** 2022-11-16

**Authors:** Sarah M. Halcomb, Arianne Johnson, S. Lena Kang-Birken

**Affiliations:** 1 Department of Pharmacy Services, Santa Barbara Cottage Hospital, Santa Barbara, California; 2 Cottage Health Research Institute, Santa Barbara Cottage Hospital, Santa Barbara, California; 3 Department of Pharmacy Practice, University of the Pacific, Thomas J. Long School of Pharmacy, Stockton, California

## Abstract

**Objective:**

To evaluate oral antibiotic prescribing for common infections at hospital discharge before and after implementation of a pharmacist-driven transitions-of-care (TOC) program.

**Design:**

Single-center before-and-after study.

**Setting:**

Acute-care, academic, community hospital in Santa Barbara, California.

**Patients:**

Eligible adult patients prescribed oral antibiotics at hospital discharge for community-acquired pneumonia, skin and soft-tissue infections, and urinary tract infections between September 2019 and December 2019 (preimplementation period) and between March 2021 and May 2021 (postimplementation period).

**Intervention:**

Antimicrobial stewardship–initiated, department-wide, TOC program requiring all clinical pharmacists to review discharge antibiotic prescriptions in real time.

**Results:**

In total, 260 antibiotic prescriptions were assessed for appropriateness: 140 before implementation and 120 after implementation. After implementation, the number of prescriptions considered inappropriate significantly decreased by 18% (52% vs 34%; *P* = .005). Inappropriate rates decreased in all assessment categories: dosing (15% vs 2%; *P* < .001), treatment duration (42% vs 31%; *P* = .08), antibiotic selection based on infection type or microbiology (8% vs 4%; *P* = .33), and antibiotics not indicated (16% vs 10%; *P* = .18). Median total antibiotic days decreased by 1 day after implementation (10 days vs 9 days; *P* = .67), and 30-day readmission rates were similar between both phases.

**Conclusions:**

A real-time, pharmacist-driven, TOC program for oral antibiotic prescriptions had a significant impact in reducing inappropriate prescribing of antibiotics at hospital discharge for common infections. Incorporating discharge antibiotic prescription review into pharmacist daily workflow may be a sustainable approach to outpatient antimicrobial stewardship in a setting with limited resources.

Antimicrobials are powerful medications that can be used to treat life-threatening infections. However, inappropriate prescribing can result in negative consequences such as emergence of antibiotic resistance and adverse reactions. Antibiotic resistance developing from inappropriate antibiotic use has been identified as one of the greatest public health threats, as well as increased risk of *Clostridioides difficile* infections.^1^ According to the Centers for Disease Control and Prevention (CDC), ∼30% of all antibiotics prescribed in the United States hospital setting are unnecessary or suboptimal, with 20% of those patients subsequently experiencing adverse effects.^1^ The most common inappropriate prescribing patterns have been related to excessive treatment duration, inappropriate antibiotic selection, incorrect doses, and conditions not warranting antibiotic treatment.^2,3^ Antimicrobial stewardship efforts have helped reduce antibiotic days of therapy and the associated risks during hospitalization, but an opportunity exists to expand these efforts to the community setting. As part of a multifaceted approach, the CDC “Core Elements of Hospital Antibiotic Stewardship Programs, 2019” recommends that antimicrobial stewardship programs (ASPs) also assess how often patients are discharged on the correct antibiotics for the appropriate duration.^1^

Recent studies have shown that many hospital-initiated antibiotic courses are completed after hospital discharge.^2,4–6^ Furthermore, community-acquired pneumonia (CAP), skin and soft-tissue infection (SSTI), and urinary tract infection (UTI) account for most inappropriate prescriptions.^2,6–8^ Therefore, assessing current prescribing practices and optimizing antibiotic discharge prescriptions for these infections could be an effective intervention in antimicrobial stewardship. Based on evaluation of our hospital discharge antibiotic prescriptions, we identified a need to optimize oral antibiotic prescriptions for common infections. Although antimicrobial stewardship needs continue to grow, many acute-care facilities are ill-equipped to support all the recommended initiatives due to inadequate resources among antimicrobial stewardship pharmacists, antimicrobial stewardship physicians, and transitions-of-care (TOC) pharmacists. We implemented a pharmacy department–wide protocol for real-time assessment of oral discharge antibiotic prescriptions, and we evaluated its impact on appropriate antibiotic usage for CAP, SSTI, and UTI at hospital discharge.

## Methods

In this single-center, quasi-experimental study, we compared appropriate antibiotic usage at hospital discharge before and after implementation of a department-wide program requiring all pharmacists to conduct real-time assessment of antibiotic prescriptions filled through the hospital-operated outpatient pharmacy. The study was approved by the organization’s institutional review board as a quality improvement project. The study site is a 519-bed, acute-care, community-based, teaching hospital and level 1 trauma center, and >50% of all discharge prescriptions are typically filled at the hospital outpatient pharmacy. The outpatient pharmacist provides medication reconciliation and discharge counseling upon the patient’s request. However, due to staffing limitations, pharmacists, inpatient or outpatient do not routinely conduct critical assessment of all discharge prescriptions.

### Intervention

The antimicrobial stewardship team developed a TOC policy requiring inpatient pharmacists to review all oral discharge antibiotic prescriptions sent to the hospital-operated outpatient pharmacy in real time. This department-wide TOC policy was approved by the antibiotic subcommittee of pharmacy and therapeutics committee and by the chief medical officer. Informational notification was sent to the prescribers regarding the new policy for inpatient pharmacists to review discharge antibiotic prescriptions being sent to the hospital-operated outpatient pharmacy. The antimicrobial stewardship pharmacist provided education to all inpatient pharmacists on the TOC policy, institutional guidelines adapted from the Infectious Diseases Society of America treatment guidelines, and clinical parameters to determine the appropriateness of the prescriptions.^9–11^ To further promote consistency, a recommended “duration of therapy” handout was created. Discharge prescriptions were configured by the information technology team to show as a stat order on the inpatient pharmacist’s order-verification queue. The pharmacist was responsible for reviewing the patient’s hospitalization medical record to verify the indication and overall appropriateness of the discharge prescription. If intervention was required, the pharmacist contacted the prescribing physician to make a recommendation or to obtain clarification. Once verified, the prescription was sent electronically to the hospital outpatient pharmacy.

### Patient population

The patients who were prescribed 1 or more oral antibiotics at hospital discharge were identified through the prescriptions filled at the hospital outpatient pharmacy during 2 phases: between September 16, 2019, and December 16, 2019 (preimplementation period) and between March 1, 2021, and May 31, 2021 (postimplementation period). Subsequently, adult patients discharged with oral antibiotic prescriptions for primary indications of CAP, SSTI, and UTI were eligible for inclusion. We excluded patients who were aged <18 years; those admitted to the emergency department only; those diagnosed with endocarditis, osteomyelitis, central nervous system infections or infections with nonbacterial pathogens; those who left the hospital against medical advice; patients discharged with parenteral antimicrobials; and patients receiving chronic immunosuppressive therapy or antibiotics for long-term suppression or prophylaxis. For quasi-random selection, the first 5 consecutive prescriptions were included for analysis, the next 5 consecutive prescriptions were excluded, and the process was repeated.

### Pre- and postimplementation assessments

The prescriptions were assessed for appropriateness in the following 4 areas: antibiotic dose, treatment duration, antibiotic selection, and need for antibiotic at discharge. Appropriate dosing was based on indication and dosing recommendations listed in Lexicomp (Lexi-Comp, Hudson, OH) and the institutional guidelines. Prescriptions were defined as having an appropriate duration if the total number of days of antibiotics, including the days during hospitalization, fell within 1 day of the recommended duration based on the guidelines and based on clinical parameters including vital signs, resolution of symptoms, white blood cell count, procalcitonin, C-reactive protein, lactate, urinalysis, imaging, and microbiology results. Appropriateness of antibiotic selection was determined based on approved indications, institutional treatment guidelines, allergies, local antibiogram, or any antibiotic exhibiting susceptibility on microbiological results. Finally, the ongoing need for antibiotics at the time of discharge was based on the total number of days of antibiotics and patient’s clinical response during hospitalization. Discharge prescriptions deemed excessive duration of therapy in the absence of any clinical reason were considered inappropriate. In addition, asymptomatic bacteriuria was considered an inappropriate indication for antibiotics. The prescription was assessed for each category of inappropriateness; however, the prescription was counted as inappropriate only once. Along with discharge antibiotic prescription data, patient demographics, comorbidities, and 30-day readmission data were collected.

### Outcomes

Our primary objective was to compare pre- and postimplementation inappropriate antibiotic prescribing rates for CAP, SSTI, and UTI indications. Secondary objectives included comparing the types of prescribing pattern change (dosage, duration, selection, and need for antibiotic) and 30-day readmission rates.

### Statistical analysis

To compare pre- and postimplementation data, statistical analysis was performed using R version 3.5.1 software (R Foundation, Vienna, Austria). Prescription inappropriateness for overall prescribing, dosage, duration, selection, need at time of discharge, and 30-day readmission were compared using the χ^2^ test of homogeneity. Post-hoc analyses using the Bonferroni correction were conducted to identify which category significantly changed in infection types. The Mann-Whitney *U* test was used to test for significance in overall antibiotic days of therapy.

## Results

### Patient characteristics

In total, 861 prescriptions identified during the preimplementation phase were filtered to include indications for CAP, SSTI, and UTI and removal of those meeting the exclusion criteria resulted in 280 prescriptions included in this study. Subsequent quasi-randomization resulted in 140 prescriptions for 140 patients. During the postimplementation phase, 619 prescriptions were identified, among which 225 prescriptions met the inclusion criteria. Finally, 120 prescriptions from 113 patients were randomly selected. Baseline patient characteristics overall were similar between groups with a few differences. The patients in the preimplementation group were older than those in the postimplementation group, with the mean ages of 66 and 60 years, respectively (Table 1).

**Table 1.** Patient Characteristics

Characteristic	Before Implementation (N = 140)	After Implementation (N = 113)	*P* Value
**Age, years**			
Range	21–96	18–95	
Mean (SD)	65.8 (18.2)	59.9 (19.5)	<.001
**Sex**			
Male, no. (%)	72 (51)	52 (46)	.767
Female, no.	68 (49)	61 (54)	
**Comorbidities**			
Diabetes mellitus	31	23	.730
Obesity	9	5	.488
AKI or CKD	26	13	.122
Liver disease	15	11	.799
CVA/TIA	14	11	.944
Myocardial infarction or congestive heart failure	12	7	.476
COPD	8	8	.657
Immunocompromised	5	3	.679
**Infection type, no. (%)**			
CAP	40 (29)	38 (34)	.387
SSTI	44 (31)	36 (32)	.942
UTI	56 (40)	39 (34)	.370

Note. SD, standard deviation; AKI, acute kidney injury; CKD, chronic kidney disease; CVA, cerebrovascular accident; TIA, transient ischemic attack; CAP, community-acquired pneumonia; COPD, Chronic obstructive pulmonary disease; SSTI, skin and soft-tissue infection; UTI, urinary tract infection.

### Pre- and postimplementation analyses

**Fig. 1.**
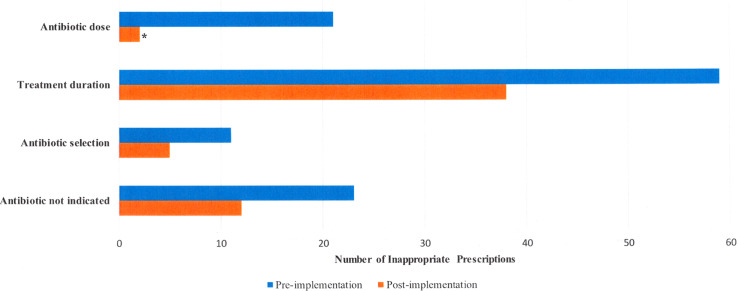
Summary of inappropriate prescribing patterns of discharge antibiotic prescriptions: before and after implementation of a pharmacist-led transitions-of-care program. *Statistical significance.

Prior to implementation, excessive treatment duration (42%) was the most common reason for inappropriateness, followed by no indication for antibiotic (16%), incorrect dose (15%), and incorrect antibiotic selection (8%) (Table 2). After the real-time pharmacist assessment and intervention implementation, the total number of antibiotic prescriptions considered inappropriate overall decreased significantly in comparison to the preimplementation period (52% vs 34%; *P* = .005). Overall rates of inappropriate prescriptions decreased in all assessment categories of dosage, treatment duration, antibiotic selection, and continued need for antibiotic at the time of discharge (Fig. 1). Notably, number of prescriptions with inappropriate dose decreased significantly by 13% (*P* < .001). Common reasons for dosing errors were related to not adjusting the dose based on patient’s renal function and selecting incorrect dose for the infection type. Excessive treatment duration showed a downward trend overall, but there was a significant reduction in the number of inappropriate prescriptions for SSTI after implementation of the TOC policy (57% vs 15%; *P* < .001). The median total antibiotic days decreased by 1 day after implementation of the TOC policy (Table 3). Most antibiotic treatment courses were completed in the outpatient setting with approximately 60% of antimicrobial exposure occurring after hospital discharge.

**Table 2.** Comparison of Inappropriate Prescribing Patterns of Discharge Antibiotic Prescriptions

Variable	Before Implementation (N = 140), No. (%)	After Implementation (N = 120), No. (%)	*P* Value
Overall prescriptions considered inappropriate	73 (52)	41 (34)	.005
Dosed inappropriately	21 (15)	2 (2)	<.001
Inappropriate duration	59 (42)	38 (31)	.08
Excessive treatment duration by infection type	CAP: 16 of 40 (40)	CAP: 20 of 40 (50)	.37
	SSTI: 25 of 44 (57)	SSTI: 6 of 40 (15)	<.001
	UTI: 18 of 56 (32)	UTI: 12 of 40 (30)	.82
Inappropriate selection^a^	11 (8)	5 (4)	.33
Antibiotic not indicated at discharge	23 (16)	12 (10)	.18

Note. CAP, community-acquired pneumonia; SSTI, skin and soft-tissue infection; UTI, urinary tract infection.

a Based on indication or microbiology results.

**Table 3.** Overall Antibiotic Days of Therapy

Variable	Before Implementation, Median (IQR)	After Implementation, Median (IQR)	*P* Value
Total antibiotic days	10 (7–12)	9 (7–12)	.67
Inpatient antibiotic days	4 (2–5)	3 (2–4)	.04
Outpatient antibiotic days	6 (4–8)	6 (4–9)	.94

Note. IQR, interquartile range.

Regarding selection of discharge antibiotics, amoxicillin-clavulanate, sulfamethoxazole-trimethoprim, cephalosporins, and fluoroquinolones were the most-prescribed agents in both periods. Notably, both levofloxacin and ciprofloxacin prescriptions decreased after implementation by approximately 40% and 50%, respectively. Positive microbiology data were available in approximately half of the patients in both study periods to assist in antibiotic selection (58% and 47%; *P* = .09). The most isolated organisms were *Escherichia coli,* methicillin-susceptible *Staphylococcus aureus,* and *Streptococcus* spp. Based on available microbiology data, an appropriate antibiotic was chosen most of the time in both the pre- and postimplementation periods (89% and 91%, respectively). In 8 cases, the appropriate antibiotics were selected based on the susceptibility report but were not de-escalated. Inappropriate antibiotic selection was often due to a culture result returning after the patient was discharged. Continuing antibiotics after completion of therapy and achieving clinical resolution during hospitalization were the primary reasons for patients not needing antibiotic prescription at discharge.

Overall, 30-day readmission rates were comparable in the pre- and postimplementation groups (10% and 12%, respectively; *P* = .547), among whom 6 patients (2 before and 4 after implementation; *P* =.273) were readmitted for reasons related to the previous discharge conditions. *C. difficile* was not reported among these subgroups.

## Discussion

Despite improved antimicrobial use through stewardship interventions in the inpatient setting, recent studies have estimated that 50%–70% of antibiotic prescriptions upon hospital discharge are inappropriate.^2,3^ Excessive treatment duration was frequently reported with approximately 60% of the treatment course occurring outside the hospital. Antimicrobial stewardship is needed during patient transitions from hospitals to outpatient settings.^5,7,12^

In this quasi-experimental study of oral discharge antibiotic prescriptions for common infections requiring hospitalization at a community hospital, we demonstrated a successful implementation of a pharmacist-led TOC program. After the implementation of real-time pharmacist assessment and intervention, the inappropriate prescription rate significantly decreased by 18%. The number of inappropriate prescriptions decreased across all subcategories of dosing, treatment duration, antibiotic selection, and need for antibiotic at discharge. The most significant impact was in antibiotic dosing, which is not surprising given that pharmacists have specialized knowledge to effectively review patient information and to determine appropriate doses. Excessive treatment duration was the most common reason for inappropriate prescribing in both study phases. This finding is similar to the findings of Vaughn et al,^5^ who assessed discharge prescriptions for CAP or UTI in a multihospital cohort. Overall, 49% of prescriptions were identified as antibiotic overuse, among which the majority were due to excess duration (63% for CAP and 44% for UTI).^5^ Interestingly, we noted a slight increase in the number of prescriptions with inappropriate treatment durations for CAP during the postimplementation period (40% vs 50%; *P* = .37). This finding may be related to empiric treatment of bacterial superinfections in the setting of coronavirus disease 2019 (COVID-19). Most prescriptions were appropriately selected. However, stewardship opportunities were identified for timely de-escalation of therapy based on the susceptibility during hospitalization as well as follow-up of microbiology results that are reported after patient discharge. Readmission rates remained similar, suggesting that improved prescribing patterns, including reduction in the total antibiotic days, did not affect patient safety. Ultimately, these improvements align with national goals to reduce inappropriate prescribing to combat the emergence of antibiotic resistance without further complications to patients.^13^

A limited number of studies have shown the impact of discharge antibiotic prescriptions stewardship by specialty-trained pharmacists.^8,14–17^ In a retrospective descriptive study of 1,100 adult patients at high risk for readmission and mortality, TOC pharmacists intervened in 298 discharge antimicrobial medications.^14^ Common intervention types were dosing (30%) and treatment duration (25%), most of which were accepted by the discharging prescribers. Parsels et al^15^ evaluated the impact of an antimicrobial stewardship team consisting of a full-time adult infectious disease (ID) pharmacist, a full-time pediatric ID pharmacist, a pharmacy school-funded ID pharmacist faculty, and a postgraduate year ID pharmacy resident on discharge oral antimicrobial prescriptions in a retrospective, single-center study. Among 438 interventions by the antimicrobial stewardship team, a significant impact was demonstrated in the median number of antimicrobial days, which decreased from 8 days (range, 5–10) to 4 days (range, 0–5.5). Despite the positive impact demonstrated by the specialty-trained pharmacists, limited resources have been mentioned in the literature. In a retrospective audit of 236 discharge antibiotic prescriptions at a 300-bed regional hospital, appropriateness of dosing (odds ratio [OR], 5.6; 95% confidence interval [CI], 1.9–9.2), microbiological specimens (OR, 4.3; 95% CI, 1.6–11.6), and targeted antibiotic selection (OR, 2.8; 95% CI, 1.8–6.2) were more likely when an antimicrobial stewardship team of an ID physician and a dedicated antimicrobial stewardship pharmacist was involved.^16^ However, due to limitations in the existing resources, only 18% of discharge antibiotic prescriptions were reviewed during the study period. Barnett et al^8^ reported an opportunity for antimicrobial stewardship intervention at hospital discharge by 1 or more antimicrobial stewardship members (eg, ID physician, fellow, and pharmacist) in a retrospective cohort study at a small Veterans’ Affairs Hospital.^8^ However, the antimicrobial stewardship reviews occurred only twice weekly due to limited resources. In a multicenter, quality improvement study of 800 patients, utilizing pharmacists to proactively identify soon to be discharged patients and to create optimal oral discharge prescriptions for final approval provided effective TOC at hospital discharge.^17^ However, this practice model requires multiple clinical pharmacists who are already integrated within the medical teams. There is a critical need for a practical approach to stewardship, especially at a community hospital with limited resources. Our TOC policy leveraging all inpatient pharmacists was unique in expanding antimicrobial stewardship services during hospital discharge without requiring additional resources. A mean of 7 prescriptions each day required additional dedicated time to review and intervene. In place of having one or more specialty-trained TOC or antimicrobial stewardship pharmacists, training all pharmacists and incorporating antimicrobial stewardship into their daily workflow results in continuity in patient care and sustainability of a successful program.

Given the nature of the retrospective review at a single center, this study had several limitations. We lacked the opportunity or documentation to further clarify specific prescribing by physicians during the preimplementation period. Implementation of the policy was delayed due to the COVID-19 pandemic. As such, the postimplementation analysis period could not be matched seasonally with the preimplementation period. Prescribing behavior may have been altered due to the unfolding COVID-19 pandemic. However, overall antibiotic susceptibility for common pathogens remained stable, and similar antibiotics were prescribed during the pre- and postintervention periods. Finally, many interventions were incompletely documented, which may have been due to the staffing shortages and frequent changes in workflow during COVID-19. Due to the lack of documentation, we may have missed prescriptions that were discontinued based on pharmacist recommendation as unnecessary at hospital discharge.

In summary, implementation of a pharmacy department–wide, real-time assessment of discharge antibiotic prescriptions had a significant impact on reducing the number of inappropriate prescriptions for common infections at hospital discharge. This study provides an example of a practical and sustainable TOC program leveraging existing pharmacist staff that can be implemented at other community hospitals with limited resources.

## References

[1] The core elements of hospital antibiotic stewardship programs, 2019. Centers for Disease Control and Prevention website. https://www.cdc.gov/antibiotic-use/healthcare/pdfs/hospital-core-elements-H.pdf. Published in 2019. Accessed August 1, 2019.

[2] Yogo N , Haas MK , Knepper BC , Burman WJ , Mehler PS , Jenkins TC. Antibiotic prescribing at the transition from hospitalization to discharge: a target for antibiotic stewardship. Infect Control Hosp Epidemiol 2015;36:474–478. 25782905 10.1017/ice.2014.85 PMC4841620

[3] Scarpato SJ , Timko DR , Cluzet VC , et al. An evaluation of antibiotic prescribing practices upon hospital discharge. Infect Control Hosp Epidemiol 2017;38:353–355. 27890038 10.1017/ice.2016.276

[4] Feller J , Lund BC , Perencevich EN , et al. Postdischarge oral antimicrobial use among hospitalized patients across an integrated national healthcare network. Clin Microbiol Infect 2020;26:327–332. 31600582 10.1016/j.cmi.2019.09.016

[5] Vaughn VM , Gandhi TN , Chopra V , et al. Antibiotic overuse after hospital discharge: a multihospital cohort study. Clin Infect Dis 2021;73:e4499–e4506. 32918077 10.1093/cid/ciaa1372 PMC7947015

[6] Conner M , Harris W , Bomkamp J. ADD it up: an evaluation of antibiotic duration at hospital discharge at a community hospital. Open Forum Infect Dis 2021;8:ofab399. 34631927 10.1093/ofid/ofab399 PMC8496735

[7] Yogo N , Shihadeh K , Young H , et al. Intervention to reduce broad-spectrum antibiotics and treatment durations prescribed at the time of hospital discharge: a novel stewardship approach. Infect Control Hosp Epidemiol 2017;38:534–541. 28260538 10.1017/ice.2017.10 PMC5612407

[8] Barnett SG , Lata P , Kavalier M , Crnich C , Balasubramanian P. Antibiotic assessment at hospital discharge—room for stewardship intervention. Infect Control Hosp Epidemiol 2020;41:209–211. 31779728 10.1017/ice.2019.332

[9] Metlay JP , Waterer GW , Long AC , et al. Diagnosis and treatment of adults with community-acquired pneumonia: an official clinical practice guideline of the American Thoracic Society and Infectious Diseases Society of America. Am J Respir Crit Care Med 2019;200:e45–e67. 31573350 10.1164/rccm.201908-1581ST PMC6812437

[10] Stevens DL , Bisno AL , Chambers HF , et al. Practice guidelines for the diagnosis and management of skin and soft-tissue infections: 2014 update by the Infectious Diseases Society of America. Clin Infect Dis 2014;59:e10–e52. 24973422 10.1093/cid/ciu444

[11] Gupta K , Hooton TM , Naber KG , et al. International clinical practice guidelines for the treatment of acute uncomplicated cystitis and pyelonephritis in women: a 2010 update by the Infectious Diseases Society of America and the European Society for Microbiology and Infectious Diseases. Clin Infect Dis 2011;52:e103–e120. 21292654 10.1093/cid/ciq257

[12] Brower KI , Hecke A , Mangino JE , Gerlach AT , Goff DA. Duration of antibiotic therapy for general medicine and general surgery patients throughout transitions of care: an antibiotic stewardship opportunity for noninfectious disease pharmacists. Hosp Pharm 2021;56:532–536. 34720157 10.1177/0018578720928265 PMC8554605

[13] National Action Plan for Combating Antibiotic-Resistant Bacteria, 2020–2025. Office of the Assistant Secretary for Planning and Evaluation website. https://aspe.hhs.gov/reports/national-action-plan-combating-antibiotic-resistant-bacteria-2020–2025. Published 2020. Accessed December 1, 2020.

[14] Leja N , Collins CD , Duker J. Antimicrobial stewardship by transitions-of-care pharmacists at hospital discharge. Hosp Pharm 2021;56:714–717. 34732928 10.1177/0018578720951170 PMC8559033

[15] Parsels KA , Kufel WD , Burgess J , et al. Hospital discharge: an opportune time for antimicrobial stewardship. Ann Pharmacother 2022;56:869–877. 34738475 10.1177/10600280211052677

[16] Chavada R , Davey J , O’Connor L , Tong D. ‘Careful goodbye at the door’: is there role for antimicrobial stewardship interventions for antimicrobial therapy prescribed on hospital discharge? BMC Infect Dis 2018;18:225. 29769028 10.1186/s12879-018-3147-0 PMC5956737

[17] Mercuro NJ , Medler CJ , Kenney RM , et al. Pharmacist-driven transitions of care practice model for prescribing oral antimicrobials at hospital discharge. JAMA Netw Open 2022;5: e2211331. 35536577 10.1001/jamanetworkopen.2022.11331 PMC9092199

